# MicroRNA miR-495 regulates the development of Hepatocellular Carcinoma by targeting C1q/tumor necrosis factor-related protein-3 (CTRP3)

**DOI:** 10.1080/21655979.2021.1973878

**Published:** 2021-09-13

**Authors:** Ruiguang Zhang, Chunxia Guo, Ting Liu, Wenting Li, Xiliu Chen

**Affiliations:** a Cancer Center, Union Hospital, Tongji Medical College, Huazhong University of Science and Technology, Wuhan City Province, Hubei, China; b Department of Infectious Diseases, Union Hospital, Tongji Medical College, Huazhong University of Science and Technology, Wuhan City Province, Hubei, China; c Department of Infectious Disease, the First Affiliated Hospital of USTC, Division of Life Sciences and Medicine, University of Science and Technology of China, Hefei, Anhui, P.R. China

**Keywords:** Mir-495, cell cycle, senescence, ctrp3

## Abstract

Hepatocellular carcinoma (HCC) represents a type of lethal cancer in the world and its treatment options produce limited and unsatisfactory effectiveness. MicroRNAs (miRNAs) that play critical roles in tumorigenesis have shown promising clinical therapeutic potential. Here, we reported that miRNA-495 (miR-495) plays important roles in inhibiting HCC cell growth via its regulation of cell-cycle progression as well as senescence. MiR-495 showed low levels in human HCC tissues and cells. Overexpressing miR-495 in HCC cells caused strong cell growth inhibition, which results from cell-cycle arrest and senescence. CTRP3 functioned as a possible target of miR-495 in HCC cells by bioinformatics prediction and biological assay. By inhibiting the expression of CTRP3 with siRNA, HCC cells also showed similar growth inhibition as miR-495 overexpression. The re-expression of CTRP3 in HCC cells with high-level miR-495 abolished miR-495 and caused cell growth inhibition. These results strongly suggested that CTRP3 was the functional target that weakened the effects of miR-495 in HCC cells. The in vivo experiment demonstrated miR-495 overexpression had great therapeutic effects on HCC in xenograft. Above all, this research revealed that miR-495 is essential in suppressing HCC growth, and its application serves as a promising strategy for HCC treatment.

## Introduction

Hepatocellular carcinoma (HCC) with highly aggressive features ranks as one of the top five frequently encountered malignancy globally [1]. HCC has badly threatened human health because of its increasing incidence, high recurrence rate and mortality rate [2]. HCC incidence has tripled since recent 30 years due to the popularization of obesity and obesity-caused diseases. Now, some clinical treatments, including surgical resection, chemotherapy, radiation therapy and liver transplantation, help to lengthen the survival time of HCC patients [3]. However, the current survival rate is still very low. Given the limited success of HCC treatment, continuous works are essential for developing efficient therapy strategies, including the identification of effective treatment targets and the research on molecular pathways in HCC treatment. Gene therapy has drawn extensive attention in recent years and has shown high efficiency, specificity and security in clinical practice [4].

MicroRNAs (miRNAs; approximately 22 nucleotides), which belong to a small non-coding RNA family, can mediate the expression of target mRNAs by binding to the 3ʹ-untranslated regions (UTR), resulting in mRNAs translational repression and/or degradation [5]. Many works showed that the dysregulation of miRNAs occurred in various human tumors, and it was involved in tumorigenesis by affecting cell proliferation, differentiation, invasion, migration, senility and apoptosis [6]. Recent reports have found the downregulation of miR-495 in various cancers developed in the lung, gastric and colorectal tissues, suggesting it might be a tumor suppressor [7,8]. Unfortunately, the exact biological functions of miR-495 in HCC were still elusive.

CTRP3 is 26-KDa in weight, which represents one member of the C1q family with pivotal functions in the modulation of metabolism, inflammation and cardiovascular system [9]. Many pieces of evidence showed that CTRP3 participated in glucose and lipid metabolism and its abnormal expression was related to some diseases, like hepatic steatosis, obesity and myocardial infarction [9,10]. Recently, this protein was demonstrated to have key regulatory effects on cell growth and/or differentiation for various cells, for instance, endothelial cell, myofibroblast, osteosarcoma, Leydig cell and vascular smooth muscle cells [10,11]. Nevertheless, the knowledge about CTRP3 in HCC was very little.

We hypothesize that in HCC, miR-495 can target CTRP3 to inhibit the proliferation of HCC. To prove this hypothesis, the present work profiled levels of miR-495 and CTRP3 in HCC tissues and matched adjacent non-tumor tissues and studied their effects on the cell cycle. In vivo and in vitro studies were performed to analyze the function of miR-495 on HCC cells. Moreover, the relationship between miR-495 and CTRP3 was also explored, which contributed to the further analysis of the action mechanism.

## Materials and methods

### Preparation of clinical tissues and cells

HCC samples and matched adjacent non-tumor tissues were from Union Hospital, Tongji Medical College, Huazhong University of Science and Technology (Approval number: HB2019-0214022S). The tissues used for experiments were collected from 118 cases surgically treated from April 2011 to March 2013 and saved in liquid nitrogen. All specimens have been given a histology diagnosis following guidelines issued by the World Health Organization. The human tissues used complied with the policies of the Institutional Review Board. Our research obtained approval from Union Hospital, Tongji Medical College, Huazhong University of Science and Technology institutional review boards. Informed consent was obtained from the patients.

Human cell lines of both HCC cells (HepG2 and Huh-7) and normal hepatic cells (HL-7702 and THLE3) were outsourced from the BeNa Culture Collection (Beijing, China). The cells were kept in a wet chamber set at 37°C, with 5% CO_2_ using either a Roswell Park Memorial Institute 1640 medium (Sigma, USA) or a 10% FBS medium of Dulbecco’s Modified Eagle Medium solution (Invitrogen, USA) [12].

### Synthesis and transfection of miRNA/siRNA/pEZ-M02

The miR-495 mimic, miR-495 antagomir (ant-miR-495), nonspecific control (NC), and siRNA targeting human CTRP3 were purchased from Guangzhou RiboBio Company (Guangzhou, China). The cells were subsequently inoculated in a 6-well plate at approximately 30–40% confluence for transference preparation. Cell transfection was performed 24 h later utilizing Lipofectamine 2000 (Invitrogen, USA) set at 50 nM RNA oligonucleotides as final concentration following manufacturer’s production protocols. After 72 h transfection, further experiments were carried out. The CDS region of CTRP3 was synthesized into the pEZ-M02 vector to construct an overexpression vector for CTRP3. After cotransfection with miR-495, a rescue validation assay was performed [13].

### Cell-counting kit (CCK)-8 assay

CellTiter96 assay kit (AdipoGen, Incheon, Korea) and CCK-8 kit (Hitachi, Japan) were utilized for cell proliferation determination following the manufacturer’s instructions. Furthermore, we also used the 5-ethynyl-2′-deoxyuridine (EdU) incorporation test for further verification as per manufacturer’s instructions (Bio-Rad, USA). Then, BD Pathway 855, the automated image system was applied to capture images and analyze the results (BD Biosciences, USA). The percentage (%) of EdU-positive cells was defined in the following formula: (added EdU cells/stained DAPI cells) × 100 [14].

### Flow cytometry

After 72 h transfection, the cells were obtained via centrifugation, rinsed with Phosphate buffer saline (PBS) and added with 300 μl PBS for resuspension. Then, 700 μl 100% ethanol was added into each group for cell fixation at 4°C for 24–48 h. The cells were subsequently cleaned and stained using 100 μg/ml propidium iodide solution and 50 μg/ml RNase solution (Invitrogen, USA) at 37°C for 30 min avoiding light. Finally, flow cytometry (BD) was utilized for cell detection following the removal of cell clumps; then, CELL Quest and ModFit LT softwares were utilized to analyze the result [15].

### Senescence β-galactosidase staining

After 3 d transfection with miR-495 or NC, HepG2 and Huh-7 cell staining was performed using the Senescence β-Galactosidase Staining Kit (CST, USA) as per manufacturer’s instructions of use [16].

### Quantitative reverse transcription-PCR (RT-qPCR)

TRIzol reagent (Promega, Madison, WI, USA) was used to collect total RNA in cell and tissue samples. Then, cDNA was synthesized using RNA through a recombinant polymerase (M-MLV Reverse Transcriptase, Promega). An SYBR Green mix (Applied Biosystems, Foster, CA, USA) was employed for a real-time PCR assay with a Chromo 4 detector (Bio-Rad, USA). U6 was served as an internal reference to normalize miRNA levels, while GAPDH was used to normalize mRNA levels. Table 1 presented primer sequences for RT-qPCR. Based on 2^–ΔΔCt^ method, the relative level of the indicated gene was calculated.

**Table 1.** Primer sequences for RT-qPCR

Gene	Gene Primer sequence (5ʹ-3ʹ)
miR-495	Forward: 5ʹ- ACACTCCAGCTGGGGAAGTTGCCCATGTT −3ʹ Reverse: 5ʹ- CTCAACTGGTGTCGTGGA −3’
CTRP3	Forward: 5ʹ- TACCTTATGCACAATGGCAACA −3ʹ Forward: 5ʹ- AGCATGATTGCTGGATGTATCTG −3’
GAPDH	Forward: 5ʹ-GAGTCAACGGATTTGGTCGT-3ʹ Reverse: 5ʹ-TTGATTTTGGAGGGATCTCG-3’
U6	Forward: 5ʹ-TTGGTCTGATCTGGCACATATAC-3ʹ Reverse: 5ʹ-AAAAATATGGAGCGCTTCACG-3’

### Western blot test

The total protein in the samples was harvested using cell lysis buffer. The same amount of proteins was applied to 10% SDS-PAGE and followed by PVDF membrane transference. Membrane blocking was conducted via 5% fat-free milk, and primary antibody was used for incubation at 4°C overnight, containing CTRP3 (mouse: Abcam; human: BD Bioscience), β-actin (Santa Cruz). After the membrane was washed, the secondary antibody was supplemented for incubation for 1 h at room temperature. Finally, the membrane was treated using an ECL kit (Pierce Chemical, USA).

### Luciferase reporter assay

The genomic DNA of HepG2 cells was PCR-amplified to produce Wt-CTRP3 3ʹ UTR. After purification, the PCR products were treated by XhoI and NotI and then inserted into psiCheck-2 plasmid (Promega). Mutation construct was acquired by a KOD kit (Toyobo, Japan) for mutagenesis following its protocol. HepG2 cells were first planted in 96-well plates and then co-transfected with 200 ng/μl recombinant plasmids and 50 nM miR-495/NC. Luciferase activity was measured in each group after 48 h co-transfection using a dual-Glo luciferase kit (Promega) [17]. The sequences for the miRNAs are listed as follows: miR-495 mimics, 5ʹ-AAACAAACAUGGUGCACUUCUU-3ʹ; anti-miR-495, 5ʹ-AAGAAGUGCACCAUGUUUGUUU-3ʹ; si-CTRP3, GCAGCTCATCTATTGGCAA.

### Mouse xenograft models

The procedures of our animal experiment were based on the Guide, short for the Guide for the Care and Use of Laboratory Animals and institutional ethical review principles for animal experimentation. All animals used in the study were approved by the animal care and use committee of Union Hospital, Tongji Medical College, Huazhong University of Science and Technology. Briefly, HepG2 cells carrying miR-495 or negative control were infected using lentivirus with 24 h incubation. Then, 100 μl cell suspension at 4 × 10^6^ cells/ml was injected into the flank of BALB/C nude mice, which were female and athymic, aged 5–6 weeks old. Nude mice were randomly numbered and randomly grouped (*n* = 60 and 30 in each group). Following 7 d, a microcaliper was employed to measure tumor size every 4 days, while a function (length×width^2^)/2 was used to evaluate tumor volume. The mice were put to death on day 31 later to harvest the tumors from each group [18,19].

### Statistical analysis

Kaplan–Meier survival curves were used to compare the disease-free survival of the HCC patients. The patients were grouped into high (above-average expression) and low (below-average expression) expression groups based on miR-495 level. Each experiment was triplicated, and all obtained data were shown as mean ± standard deviation. Analysis of the results was performed using the SPSS 19.0 software. Standardized expression data were analyzed statistically for significance determination using paired or unpaired Student’s *t*-tests. The values of *P* < 0.05 were defined as statistically significant.

## Results

In this study, we hypothesized that in HCC, miR-495 can target CTRP3 to inhibit the proliferation of HCC. To prove this hypothesis, we first detected the expression difference of miRNA-495 in normal tissues and tumor tissues. Subsequently, the regulation of miR-495 on the proliferation of HCC cells was studied in vivo and in vitro, and the targeting relationship between miR-495 and CTRP3 was proved.

### Poor expression of miR-495 in HCC tumors and cell lines

**Figure 1.**
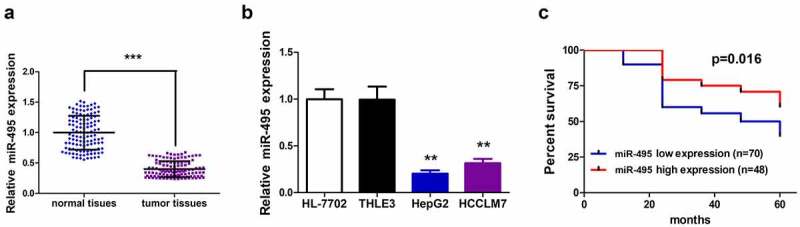
MiR-495 expression profile and Kaplan–Meier survival curves of HCC patients. (a) MiR-495 expression in tumor tissues was subjected to real-time PCR and the expression of U6 small nuclear RNA was standardized, *n* = 118. (b) MiR-495 expression in HCC cells was subjected to real-time PCR and the expression of U6 small nuclear RNA was standardized. (c) Kaplan–Meier survival curves for disease-free survival of the HCC patients. the patients were grouped to high- and low-expression groups based on mir-495 level. log-rank tests were used for data comparison. all of the experiments were repeated at least three times. **P < 0.01, ***P < 0.001

The expression profile of miR-495 in human HCC tissues and adjacent non-neoplastic tissues was detected, and RT-qPCR was performed to test its level in a total of 118 pairs of clinical tissues. The data showed that the level of miR-495 in tumor tissues was lower than that of the adjacent normal tissues (P < 0.001) (Figure 1(a)). To verify this result, miR-495 expression in two HCC cell lines (HepG2 and HCCLM7) and two normal human liver cell lines (HL-7702 and THLE3) was detected, respectively. Unsurprisingly, miR-495 was low-expressed in HepG2 and HCCLM7 cells comparing to normal cells (Figure 1(b)). Then, we used Kaplan–Meier survival curves to further examine whether the decreased miR-495 levels could predict prognosis of HCC patients. The results showed that low miR-495 level patients developed poorer disease-free survival than those of high miR-495 level (Figure 1(c); *P*= 0.016).

### miR-495 inhibited cell growth and induced senescence in HCC cell lines

**Figure 2.**
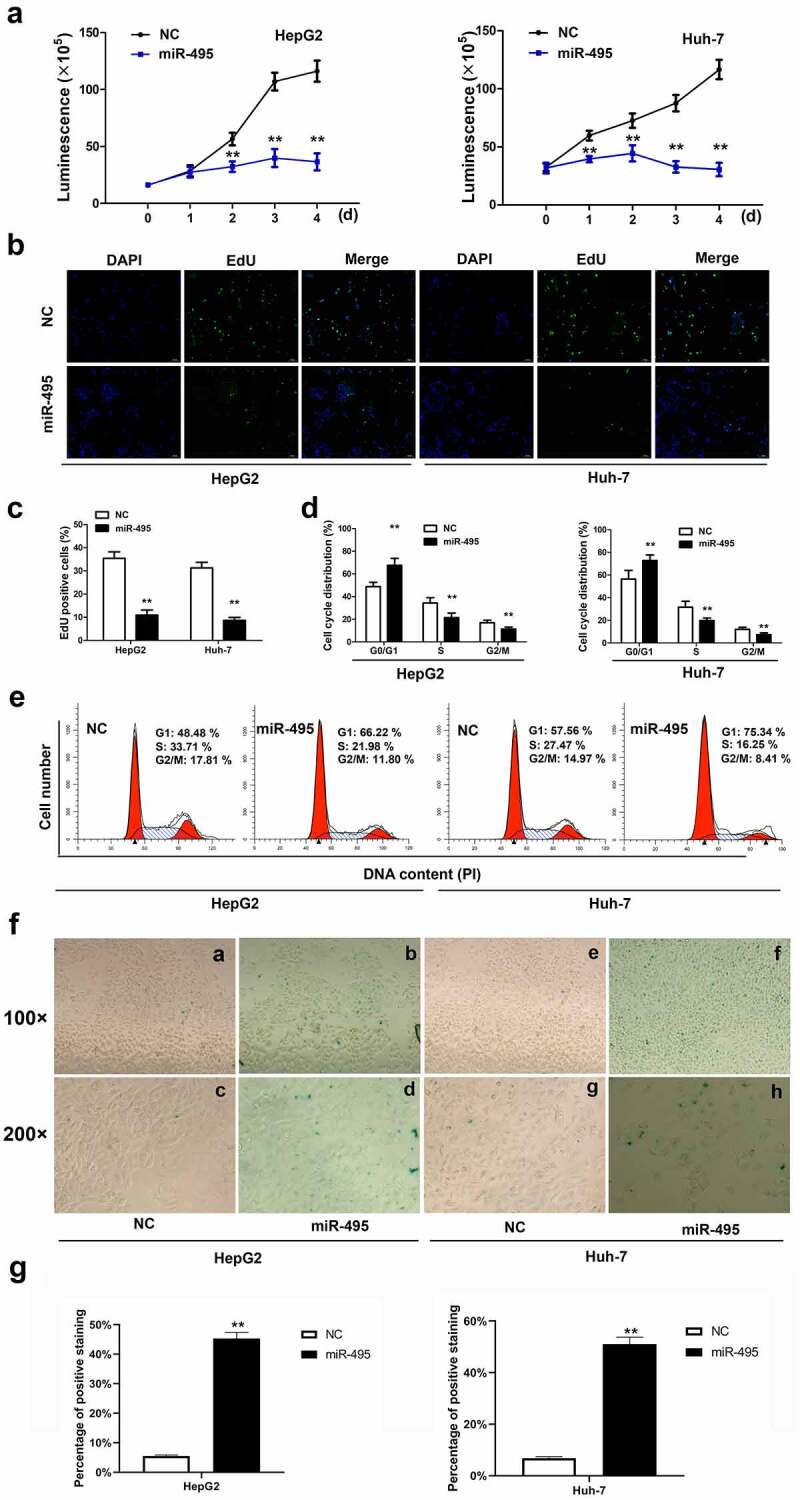
MiR-495 inhibits cell growth and induces senescence in HCC cells. (a) cell viability assays were performed for HepG2 and Huh-7 cells were transfected with miR-495 (blue line) or negative control vector (dark line) at indicated time. (b-c) The EdU incorporation assay was performed to analyze the proliferation ability of HepG2 and Huh-7 cells transfected with miR-495 or negative control vector. (d-e) Cell cycle was assessed in HepG2 and Huh-7 cells transfected with miR-495 or negative control vector by propidium iodide staining using flow cytometry. (f-g) senescence was measured by SA-β-gal staining in miR-495 transfected HCC cells. a, b, e and f displayed figures of 100× magnification. c, d, g and h displayed figures of 200× magnification depicting changes in morphology. The data were calculated as the average of triplicate values. ** represented *P* < 0.01

To detect whether miR-495 mediated HCC cell growth, the transfection of either miR-495 mimic or miR-NC was conducted into HepG2 and Huh-7 HCC cells. The results of CCK-8 assay displayed that the overexpression of miR-495 weakened cell proliferation for both two cell lines (Figure 2(a)). EdU incorporation assay testified that miR-495 inhibited cell proliferation (Figure 2(b-c)). Cell cycle analysis was performed to determine the inhibitory mechanisms of miR-495 on cell growth. Figure 2(d-e) indicated an increase in cell percentage in phase G0/G1 and a decrease in phase S following the overexpression of miR-495 compared to the negative control. Moreover, cell ratio in phase G2/M was also notably declined. As senescence is also a known mechanism of cell growth inhibition, we also examined whether miR-495 overexpression could induce senescence for HCC cells. Interestingly, upregulating miR-495 induced senescence for the two kinds of HCC cells (Figure 2 (f-g)). Therefore, the data implied that miR-495 suppresses cell growth mainly by arresting cells at the G0/G1 phase and inducing senescence.

### CTRP3 is one target of miR-495

**Figure 3.**
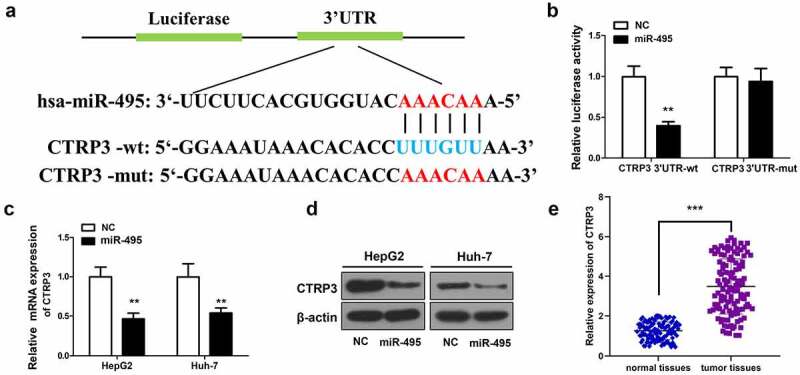
CTRP3 is a direct target of miR-495. (a) sequence alignment between miR-495 and the 3ʹ-UTR of the human CTRP3 mRNA. (b) The luciferase activities of wt/mutant CTRP3 3ʹ-UTR reporter constructs was analyzed in HCC cells transfected with miR-495 or miR-NC. (c) The mRNA levels of CTRP3 in HCC cells were analyzed using real-time PCR and standardized against β-actin. (d) CTRP3 protein levels were subjected to western blotting. (e) The mRNA levels of CTRP3 were profiled in tumor tissues and normal tissues (*n* = 20). **P < 0.01, ***P < 0.001

Next, the mechanisms of miR-495 were explored by identifying one possible downstream targets of miR-495 – CTRP3 by using TargetScan (http://www.targetscan.org/vert_71/). A binding site of miR-495 was exhibited in CTRP3 3ʹ-UTR (Figure 3(a)). CTRP3 was subsequently confirmed as a target of miR-495 in HCC cells via a luciferase reporter assay. The results presented a decrease in the reporter luciferase activity induced by overexpressing miR-495 in wild-type CTRP3 group, while no significant change in the mutant-type CTRP3 group (Figure 3(b)). In addition, both the mRNA and protein levels of CTRP3 were remarkably decreased following miR-495 overexpression (Figure 3(c-d)). Conversely, the mRNA level of CTRP3 was significantly elevated in HCC tissues (Figure 3(e)). Based on the above results, we estimated that CTRP3 acted as a target of miR-495 in HCC.

### miR-495 overexpression inhibits HCC cell growth mainly through targeting CTRP3

**Figure 4.**
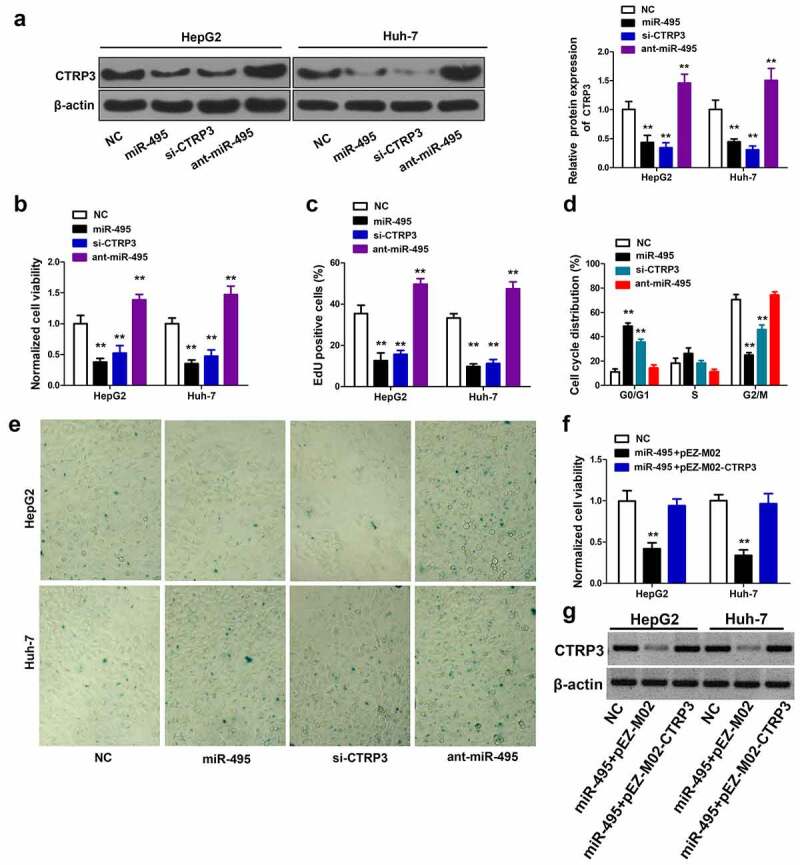
The inhibitory effect of miR-495 overexpression is through targeting CTRP3. (a) The protein levels of CTRP3 were identified using western blot assay in HCC cells transfected with indicated plasmid. (b) after indicated plasmid was transfected into HCC cells, and the cell growth was determined using CCK-8 assay. (c) the cell proliferation ability was determined using EdU incorporation assay in HCC cells transfected with indicated plasmid. (d) the cell cycle profile was measured via propidium iodide staining using flow cytometry. (e) cellular senescence was measured via SA-β-gal staining after transfection of HCC cells. (f) after HCC cells were transfected with indicated plasmid (200 ng/mL of pEZ-M02-CTRP3 or pEZ-M02; 50 nM miR-495 mimics), and the cell viability was measured by CellTiter96 assay kit. (g) CTRP3 protein expression was detected by western blot. **P < 0.01

To explore the role of CTRP3 in HCC cells, si-NC, miR-495, si-CTRP3 and ant-miR-495 were separately transfected into both HepG2 and Huh-7 cells. Figure 4(a) showed that si-CTRP3 significantly decreased CTRP3 protein levels. MiR-495 overexpression also notably inhibited CTRP3 protein expression, while ant-miR-495 strongly elevated CTRP3 protein levels. As expected, CTRP3 knockdown exhibits a similar effect as miR-495 overexpression in HCC cells. CCK8 experiment demonstrated that the CTRP3 knockdown greatly reduced cell proliferation for the two cell lines (Figure 4(b)). The EdU incorporation assay showed that CTRP3 knockdown significantly decreased EdU positive cells (Figure 4(c)). The CTRP3 knockdown also arrested cells at the G0/G1 phase and induced numerous senescent cells (Figure 4(d-e)). Moreover, the overexpression of ant-miR-495 presented an opposite effect as CTRP3 knockdown, indicating miR-495 induced cell growth inhibition is mainly through targeting CTRP3. The rescue experiments further confirmed our model, as overexpression of CTRP3 in miR-495 overexpressed cells abolished miR-495 induced cell growth inhibition (Figure 4(f-g)). Taken together, miR-495 overexpression inhibits HCC cell growth mainly through targeting CTRP3.

### miR-495 retards tumor growth in animal experiment

**Figure 5.**
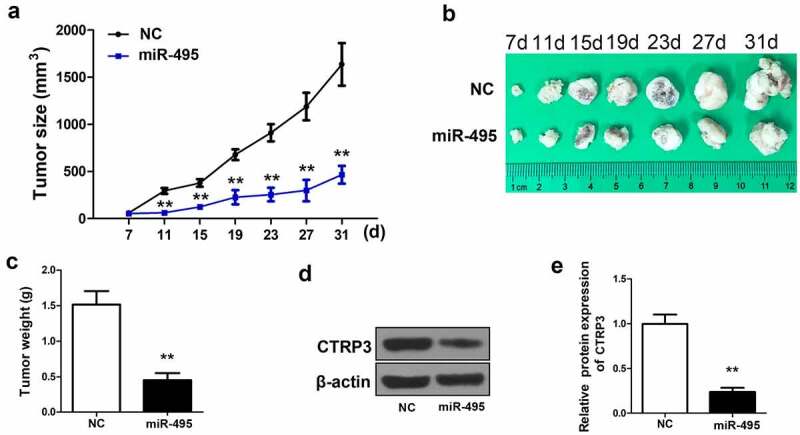
MiR-495 suppresses tumor growth in vivo. (a) A microcaliper was employed to measure tumor size every 4 days 7 d after injection, and calculated as (length×width^2^)/2 (60 BALB/C female athymic nude mice, aged 5–6 weeks,). (b) the tumor size at 7 d, 11 d, 15 d, 19 d, 23 d, 27 d and 31 d. (c) the weight of the tumors after 31 days was determined. (d) protein level of CTRP3 in tumors was determined via western blotting. (e) the protein levels of CTRP3 was quantified and shown in histogram. **P < 0.01

To clarify the inhibitory effects of miR-495 on HCC tumor growth in vivo, lentivirus carrying miR-495 or negative control was transfected with HepG2 cells and injected into the selected laboratory mice. The data showed that the miR-495 overexpressed group presented a smaller tumor size compared to the negative control group during the treatment process (Figure 5(a)-5(b)). , miR-495 was noticed that it apparently retarded tumor growth in vivo in comparison to the NC group. A significant decrease in tumor weight was also observed in miR-495 overexpressed group (Figure 5(c)). The expression of CTRP3 was further determined in the previously described tumors. More than three times the decrease of CTRP3 expression was visualized in the miR-495 overexpressed group, which was consistent with the result of cell experiment (Figure 5(d-e)). The findings indicated that miR-495 overexpression could inhibit HCC cells growth, and the inhibitory effect was mainly through targeting CTRP3 expression.

## Discussion

HCC has been considered as the fifth most aggressive solid cancer in the world, leading to approximately 600,000 deaths each year. Unfortunately, only one chemotherapy drug (sorafenib) is available in clinical for HCC treatment because of the poor understanding of the mechanisms of this cancer [20]. Therefore, more attention is to be paid to reveal the underlying mechanisms as well as new target identification of this malignancy for future treatment of this disease.

MiRNAs have drawn extensive attention in recent years, and their aberrant expression contributes to the morbidity and progression of human cancer. Increasing evidence showed that miR-495 downregulation occurred in many human cancers, whereas the overexpression of which could inhibit the growth of the tumor and cancer cells, namely lung cancer, gastric cancer and colorectal cancer [7,8]. Previous studies have shown that IGF1R was the direct target gene of miR-495 in HCC. IGF1R was up-regulated in HCC tissues and was negatively correlated with the expression level of miR-495 [21]. In this study, first, 118 pairs of clinical tissues were collected, and the mRNA levels of miR-495 were compared. We found that the level of miR-495 was largely declined in the HCC tissues by comparing with the surrounding normal tissues. The findings are in agreement with previous research, indicating that miR-495 suppressed tumors and had a low level in many human cancers [8,21,22]. Moreover, the decrease of miR-495 expression levels was greatly involved in HCC development and unfavorable prognosis. miR-495 mimic and the negative control were subsequently transfected into HCC cells. The high-level miR-495 reduced cell proliferation and growth of HCC cells, which was associated with cell cycle arrest and cellular senescence induction. This was consistent with the data on colorectal cancer, which indicated that miR-495 bound to FAM83D and arrested cell cycle at phase G1/G0 [8]. In addition, we also provide more targeted regulatory mechanisms for miR-495. It provides a basis for an in-depth and comprehensive understanding of its regulatory network.

It is recognized as an essential step to explore the roles of miR-495 in HCC pathogenesis by identifying the functional targets of miR-495. Based on bioinformatics analysis, many targets of miR-495 are predictable. CTRP3 was a potential downstream target of miR-495 at the presence of a binding site in CTRP3 3ʹ-UTR. We then experimentally confirmed that miR-495 could suppress CTRP3 expression by directly combining to 3ʹ-UTR of CTRP3. Although CTRP3 plays a central role in regulating lipid and glucose metabolism, the biological function of CTRP3 is not restricted to metabolic regulation [9]. It has been shown that CTRP3 could stimulate cell proliferation and migration and regulate expressions of connective tissue growth factor by TGF-β1, suggesting the possible function of CTRP3 in oncogenesis [9,23,24]. Studies on osteosarcoma have shown that miR-495-3p overexpression inhibits cell proliferation, migration and invasion through downregulation of CTRP3. miR-495-3p may serve as a tumor suppressor and a potential target for OS therapy [22]. Therefore, it is very necessary to measure the expression condition and function of CTRP3 in HCC. Subsequently, RT-qPCR demonstrated that the level of CTRP3l in HCC tissues was higher than that of the adjacent non-neoplastic tissues. As CTRP3 is one direct target of miR-495, miR-495 downregulation in HCC tissues resulted in the upregulation of CTRP3. Then, we further validated whether miR-495 overexpression could lead to cell cycle arrest and senescence, mainly through suppressing CTRP3 expression. Furthermore, the knockdown of CTRP3 presented similar effects as miR-495 overexpression on HCC cells. The re-expression of CTRP3 rescued the inhibitory effects as a result of overexpression of miR-495 in HCC cells. Our findings were consistent with those of previous studies.

Finally, an animal experiment presented that miR-495 prevented HCC tumor growth in mice. The MiR-495 overexpressed group presented smaller tumor size and weight compared to the negative control group. Additionally, more than three times decrease of CTRP3 expression was observed in miR-495 overexpressed group. The results in vivo were consistent with those researches in vitro, indicating that miR-495 overexpression could suppress HCC cells growth, and the inhibitory effect is mainly through targeting CTRP3 expression.

## Conclusion

This work revealed that miR-495 was low-expressed in HCC tissues, and its overexpression induced cell cycle arrest and senescence and then led to strong cell growth inhibition. The inhibitory effects following miR-495 overexpression through directly targeting the 3ʹ UTR of CTRP3 thereby blocking CTRP3 expression. The in vivo experiments showed miR-495 could remarkably inhibit HCC tumor growth, suggesting miR-495 had the possibility to be a potential therapeutic agent used for HCC therapy.

## Supplementary Material

Supplemental Material

## Data Availability

All data generated or analyzed during this study are included in this published article.

## HIGHLIGHTS

miR-495 inhibited cell growth and induced senescence in HCC cell lines.

CTRP3 is one target of miR-495.

miR-495 retarded tumor growth in animal experiments.
